# Denial attitude towards COVID-19 among general population in Saudi Arabia

**DOI:** 10.1192/j.eurpsy.2021.795

**Published:** 2021-08-13

**Authors:** S. Alsubaie, H. Alshahrani, A. Alshahrani, A. Asiri, A. Alfaifi, R. Al Ibrahim, W. Alqahtani

**Affiliations:** 1 Psychiatry, Armed Forces Hospital Southern Region, Khamys Mushait, Saudi Arabia; 2 College Of Medicine, King Khalid University, Khamys Mushait, Saudi Arabia

**Keywords:** COVID-19, Denial attitude, public health, Saudi Arabia

## Abstract

**Introduction:**

During the current crisis of COVID 19, recent studies evident that it has a huge impact on public mental health and individuals’ behavior.

**Objectives:**

Our study aimed to estimate the prevalence of high denial attitude towards the emerging pandemic of COVID 19 among the general population of Saudi Arabia.

**Methods:**

A cross-sectional online survey was conducted from April 3, 2020 to May 5, 2020. All participants (N= 1817) were asked to complete an online questionnaire survey that included socio-demographic and other variables, and Denial Attitude Questionnaire towards COVID-19 pandemic (DAQ-COVID-19).

**Results:**

High denial attitude was prevalent among 728 (40.1 %) of the participants. It was associated with old age, being married, having low educational level, working in a non-medical professions, do not have a past history of infectious diseases, spending less than one hour following COVID-19 news, satisfied with the government procedures for COVID-19, and highly depressed and anxious respondents, where p-values were 0.001, 0.019, <0.001, 0.027, <0.001, <0.001, 0.004, 0.008, and 0.026; respectively.

**Conclusions:**

About two out of five participants had high denial attitude. To our knowledge, the current study is the first study that tries to evaluate a high denial attitude during the initial COVID 19 outbreaks, especially in Saudi Arabia. However, further exploration in this field is needed. We suggest conducting such a study at the end of the current pandemic or in the second wave of the outbreak

